# Rift-inversion orogens are potential hot spots for natural H_2_ generation

**DOI:** 10.1126/sciadv.adr3418

**Published:** 2025-02-19

**Authors:** Frank Zwaan, Sascha Brune, Anne C. Glerum, Dylan A. Vasey, John B. Naliboff, Gianreto Manatschal, Eric C. Gaucher

**Affiliations:** ^1^ GFZ Helmholtz Centre for Geosciences, Potsdam, Germany.; ^2^ Department of Geosciences, University of Fribourg, Fribourg, Switzerland.; ^3^ Institute of Geosciences, University of Potsdam, Potsdam, Germany.; ^4^ Department of Earth and Climate Sciences, Tufts University, Medford, MA, USA.; ^5^ Department of Earth and Environmental Science, New Mexico Institute of Mining and Technology, Socorro, NM, USA.; ^6^ University of Strasbourg, CNRS, ENGEES, ITES UMR, 7063 Strasbourg, France.; ^7^ Lavoisier H_2_ Geoconsult, Chamonix, France.

## Abstract

Naturally occurring hydrogen gas (H_2_) represents a potential source of clean energy. A promising mechanism for large-scale natural H_2_ generation is serpentinization of exhumed mantle material. We study this serpentinization-related H_2_ generation during rifting and subsequent rift-inversion orogen development using numerical geodynamic models. Serpentinization-related H_2_ generation is best known from rifted margins and spreading ridges. However, because orogens are colder than rift environments, conditions for serpentinization and natural H_2_ generation are considerably better in orogenic settings: We find that yearly H_2_ generation capacity from serpentinization in the overriding mantle wedge during rift inversion may be up to 20 times larger than during rifting. Moreover, suitable reservoirs and seals required for economic H_2_ accumulations to form are readily available in rift-inversion orogens but are likely absent during bulk serpentinization in rift settings. Together with indications of ongoing natural H_2_ generation in the Balkans and Pyrenees, our model results provide a first-order motivation for natural H_2_ exploration in rift-inversion orogens.

## Teaser

Rift-inversion orogens are promising sites for large-scale occurrence of natural H_2_, a clean energy source for the future.

## INTRODUCTION

A key challenge for the 21st century is the development of sustainable energy sources. Molecular hydrogen gas (henceforth “H_2_”) may be one of our best alternatives to hydrocarbon-based fuels, but present-day synthetic H_2_ production is costly (*1*, *2*). However, H_2_ is also generated by a range of natural (bio)chemical processes in the lithosphere [e.g., (*3*, *4*)], and this “natural H_2_” may represent an excellent source of sustainable energy that, until recently, has been mostly overlooked e.g., (*5*–*7*).

A prominent mechanism for large-scale natural H_2_ generation is the alteration, or serpentinization, of minerals in (ultra)mafic mantle rocks when they react with liquid water (*5*, *8*). This mechanism is very efficient along rifted margins and mid-oceanic ridges that form during the divergence stage of the Wilson cycle (*9*), where mantle rocks are permanently serpentinized close to the ocean floor (*10*–*13*), but industrial extraction of natural H_2_ is likely to be uneconomic in these far offshore and deep-water environments. Onshore exploration is therefore more promising, where serpentinization-derived natural H_2_ is unequivocally sourced from mantle rocks exposed at the surface [e.g., in Oman (*14*) and New-Caledonia (*15*)] or at relatively shallow (several kilometers) depth [e.g., in Albania (*16*) and Kosovo, (*17*)].

However, because serpentinization and natural H_2_ generation is most efficient at temperatures over 200°C (*18*, *19*), and because H_2_ is quickly consumed by a range of (bio)chemical reactions near the surface (*20*, *21*), the volumes of natural H_2_ available from shallow mantle rocks most likely remain relatively small. Instead, natural H_2_ derived from mantle bodies at greater depth (>10 km) could be the best target for industrial extraction. Fossil evidence of natural H_2_ generation has been reported in the European Alps (*22*–*24*), whereas recent research from the Western Pyrenees points to the presence of natural H_2_ at the surface that is potentially being generated from mantle rocks at depth (*21*, *25*, *26*).

**Fig. 1.**
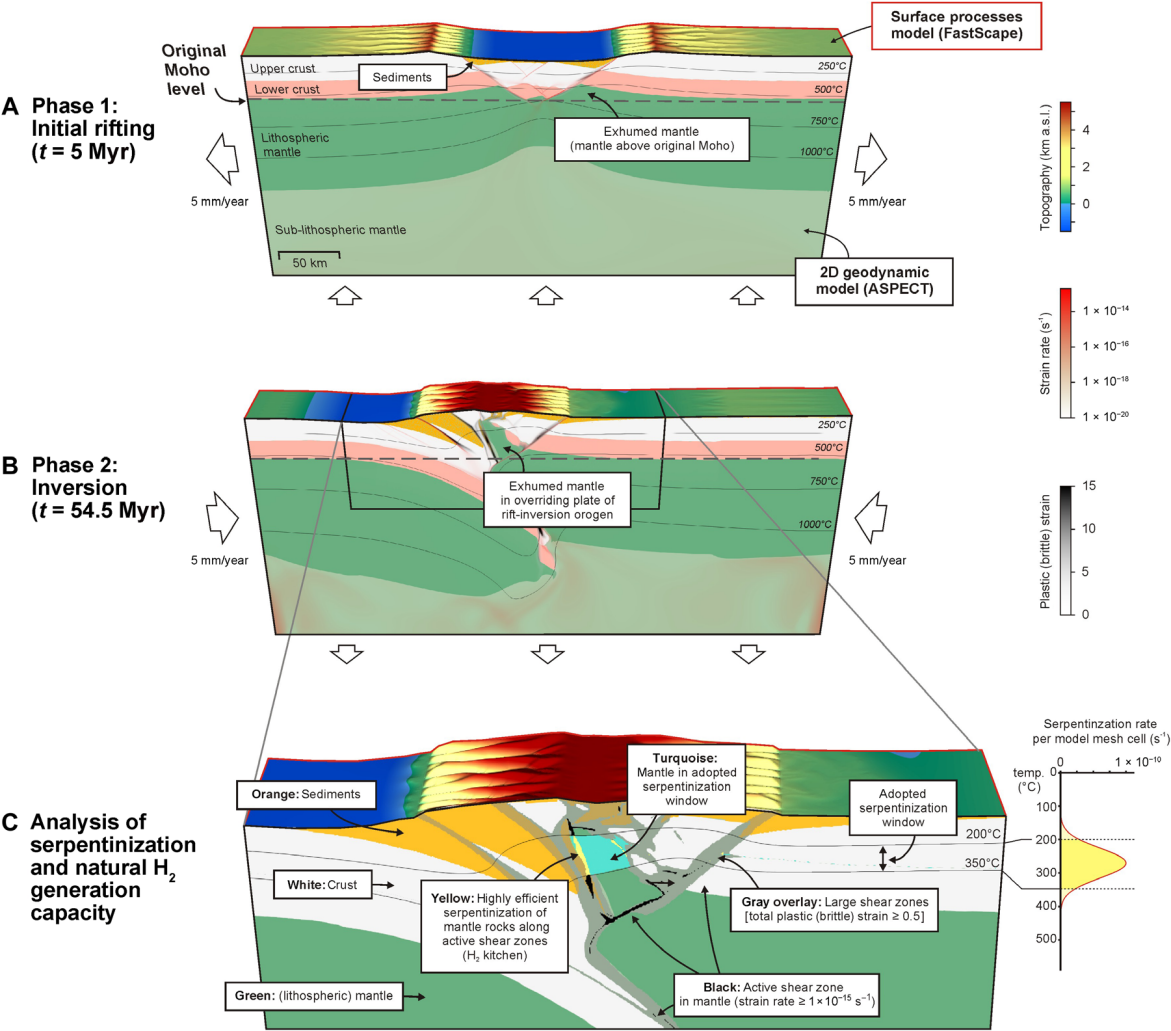
Example of a model run and definitions used for model analysis. 3D rendering of model B, with 15 Myr of initial rifting, followed by a 20 Myr of tectonic pause before 20 Myr of inversion. The ASPECT geodynamic model is 2D, whereas the FastScape surface processes model, interacting with the top boundary of the ASPECT model, provided a quasi-3D model result. See Materials and Methods for more details on the overall geodynamic modeling approach. (**A** and **B**) General model evolution, with the definition of exhumed mantle [mantle material found above the initial base of the crust (i.e., the original Moho]. (A) Model state after 5 Myr of rifting. (B) Model state toward the end of the model run (*t* = 54.5 Myr). (**C**) Definitions used for serpentinization and natural H_2_ generation analysis in rift enviromnents and in the overriding mantle wedge in subsequent rift-inversion orogens. Although serpentinization is also known to occur at higher temperatures and pressures, we adopt the 200° to 350°C temperature range (i.e., the “serpentinization window”) to conservatively visualize the H_2_ kitchen (in yellow), where efficient serpentinization occurs along large active faults, which are assumed to allow for ample water circulation. The adopted serpentinization window is based on the temperature-defined formula for serpentinization from (*12*), which we use for the actual estimation of serpentinization and associated H_2_ generation. This formula follows a bell curve that peaks within the adopted 200° to 350°C temperature range and is near zero outside of it, illustrating the validity of this temperature range for visualization purposes. See the Materials and Methods section and the supplementary data (*37*) for more details on the serpentinization and natural H_2_ generation analysis, as well as the results of various parameter tests.

The Alps and Pyrenees are rift-inversion orogens, formed by large-scale inversion of rift basins during the convergence stage of the Wilson cycle (Fig. 1) (*9*, *27*). They have in common that ultramafic mantle rocks are exhumed [here defined as being brought above the original depth of the crust-mantle boundary (Moho) by tectonic and erosional processes] and emplaced in the overriding plate [e.g., (*28*, *29*)]. This exhumation enhances the likelihood of mantle material coming into contact with liquid water (of marine, meteoric, or metamorphic origin), which is a prerequisite for serpentinization to occur, while remaining at favorable temperatures for efficient hydration. Yet, despite recent efforts (*12*, *13*), our understanding of the dynamics and timing of mantle exhumation and large-scale serpentinization processes in rift-inversion orogens, crucial to assess the associated natural H_2_ potential (*1*), remain limited, especially because these processes occur at depth.

In this context, geodynamic modeling provides a vital means of gaining insights into otherwise hidden subsurface processes, and various researchers have used numerical modeling methods to study the evolution of rift-inversion orogens [e.g., (*27*, *30*–*32*)]. Still, these studies mostly focused on crustal structures, providing very few constrains on how rift-inversion orogens may facilitate large-scale natural H_2_ generation during their evolution. Here, we present generalized numerical geodynamic models that include all key first-order processes required to describe the large-scale thermomechanical evolution of rift-inversion orogens. These models allow us to assess where and when mantle rocks may be both at favorable temperatures and in contact with water as they are exhumed below the thinning continental crust during rifting and in the overriding mantle wedge during subsequent inversion. Through this approach, we can quantify how and when tectonic processes enable large-scale serpentinization and natural H_2_ generation linked to exhumed mantle material over the course of rift-inversion orogen evolution. In addition, our modelling approach allows us to conceptually link, in time and space, the possible location of natural H_2_ generation with potential migration pathways (faults) and potential reservoirs (sediments). Together, these concepts are required to effectively characterize “hydrogen systems” in nature and to identify potential targets for natural H_2_ exploration (*21*, *33*).

## RESULTS

Here, we use the geodynamic software ASPECT [e.g., (*34*, *35*)] coupled with the surface processes code FastScape [e.g., (*36*)] to conduct numerical models of rift-inversion orogens (Fig. 1; for more details, see Materials and Methods). We systematically quantify the impact of initial rifting duration and the occurrence of a post-rift cooling phase on mantle exhumation and associated large-scale H_2_ generation during rift-inversion orogen evolution.

**Fig. 2.**
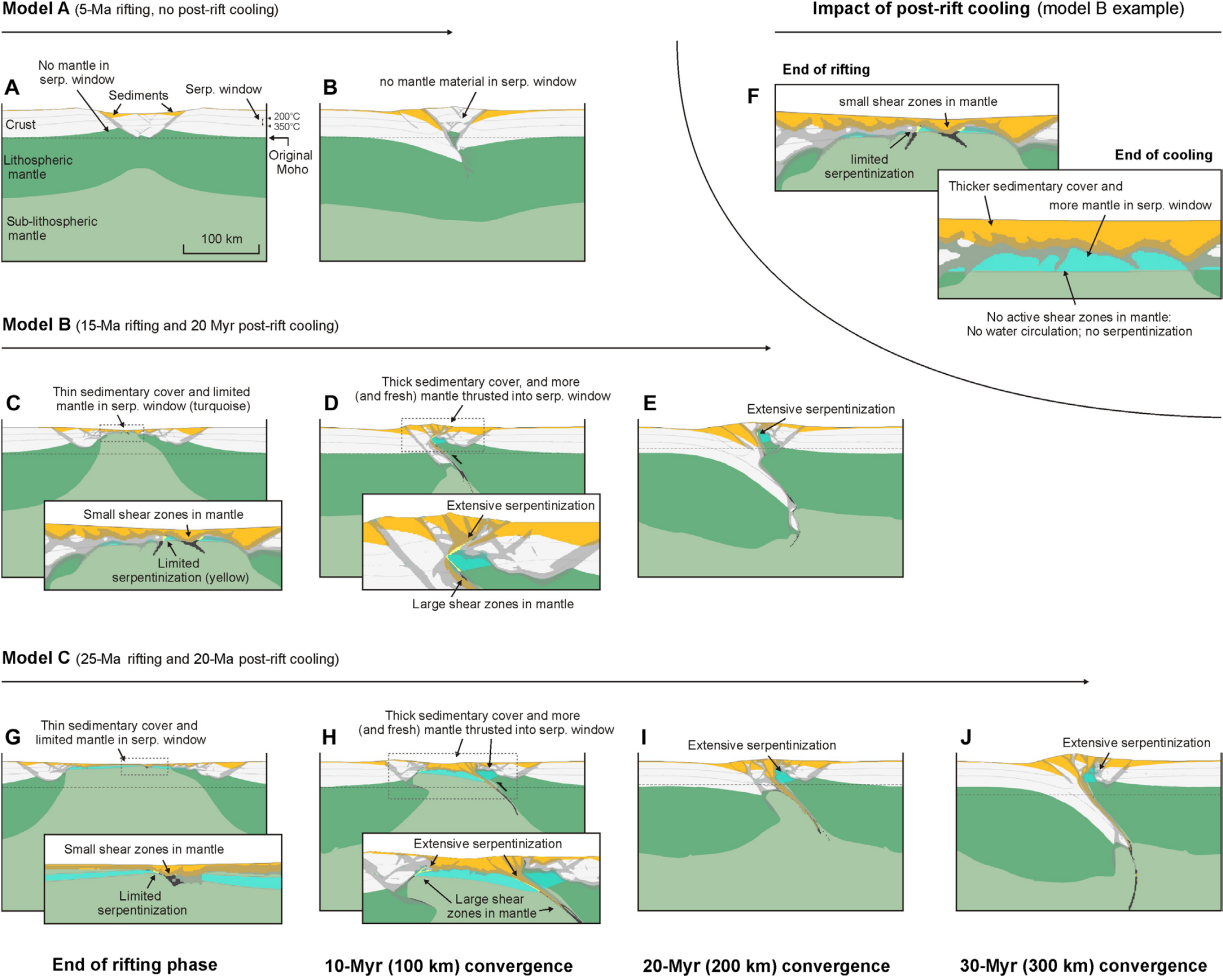
Evolution of key rift-inversion orogen styles. Results shown in (**A**) and (**B**) are from model A, in (**C**) to (**F**) are from model B, and in (**G**) to (**J**) from model C, respectively, with snapshot timing for all models as indicated below model C. We indicate the associated serpentinization as a function of rift duration and post-rift cooling before inversion, where insets in (C) and (D) and (G) and (H) are used to highlight important details. Panel (F) serves to highlight the impact of post-rift cooling in model B. Light green: sub-lithospheric mantle; dark green: lithospheric mantle; white: crust; orange: sediments; turquoise: mantle material in the adopted serpentinization window (between the 200° and 350°C isotherms), transparent gray: fault zones; black: active fault zones in mantle; yellow: areas of highly efficient serpentinization (the H_2_ kitchen). See also annotation in (A) and Fig. 1C. Limited serpentinization: up to 5 × 10^9^ kg(km year)^−1^ (during rifting); extensive serpentinization: up to 1 × 10^11^ kg(km year)^−1^ (during inversion). The results from the serpentinization and H_2_ potential analysis are presented in Fig. 3.

**Fig. 3.**
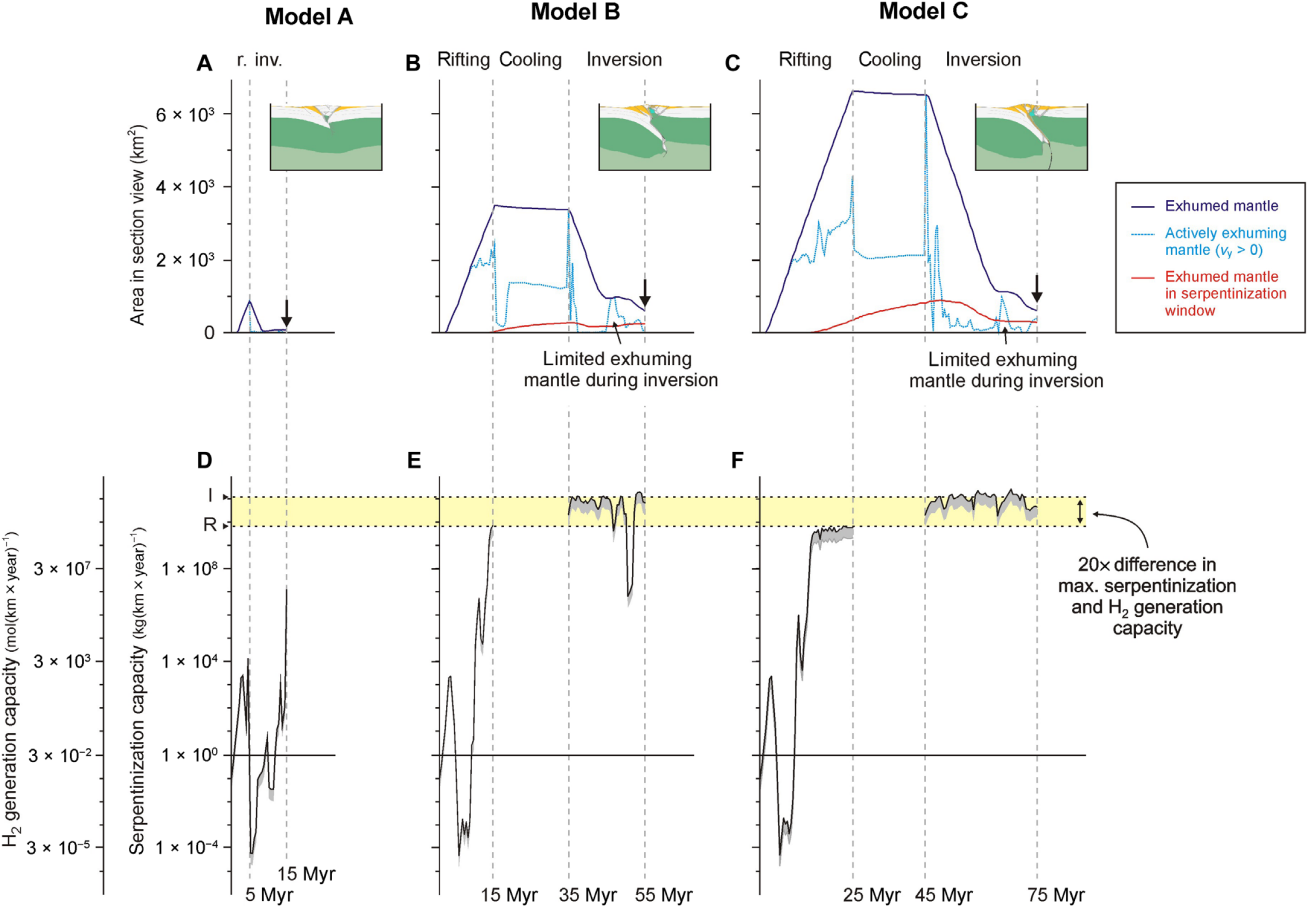
Analysis of serpentinization and natural H_2_ generation potential during the evolution of key rift-inversion orogen styles. We show results from models A, B and C, respectively (see also Fig. 2). (**A** to **C**) Total amount of exhumed mantle (dark blue), amount of actively exhuming mantle, i.e., upward vertical motion >0 m s^−1^ (turquoise), and mantle area within the adopted serpentinization window (200° to 350°C) over time (red) for each model. The timing of the model insets is indicated with a black arrow (10, 20 and 30 Myr after the start of inversion, respectively). (**D** to **F**) Serpentinization capacity and natural H_2_ generation capacity over time. Because of the 2D modeling geometry, all values are provided per along-strike unit length. Note that in these plots we assume that 1 kg of serpentinized mantle generates the maximum of 300 mmol of natural H_2_ to draw the black curves, whereas the gray area below these curves indicates the possible variation, assuming that 1 kg of serpentinized mantle may generate between 100 and 300 mmol of natural H_2_ (*11*). R: Maximum serpentinization and natural H_2_ capacity during rifting [i.e., ~5 × 10^9^ kg(km year)^−1^ and ~1.5 × 10^9^ kg(km year)^−1^, respectively]. I: Maximum serpentinization and natural H_2_ capacity during inversion [~1 × 10^11^ kg(km year)^−1^ and ~3 × 10^10^ mol(km year)^−1^, respectively]. The model results show that serpentinization and associated H_2_ generation is enhanced ~20-fold during inversion, provided that sufficient initial rifting is allowed to take place.

Our key model results are presented in Figs. 2 and 3. Throughout each model’s tectonic evolution, we quantify as a first step in two-dimensional (2D) section view the following three attributes: the area of exhumed mantle (i.e., the area of mantle material found above the pre-rift Moho depth of 35 km in our models), the area of actively exhuming mantle (i.e., exhumed mantle that is moving upward), and the area of exhumed mantle material in the “serpentinization window” (i.e., at temperatures between 200° and 350°C), in which we assume that bulk serpentinization could occur through efficient mantle hydration, if sufficient water would be available [e.g., (*18*) (Fig. 1C and Fig. 3A to C)]. The extent of the adopted serpentinization window is based on the temperature-dependent serpentinization formula from (*12*), where serpentinization efficiency follows a bell curve with its peak (i.e., bulk serpentinization) centered in the 200° to 350°C temperature range (Fig. 1C). Note that our serpentinization parametrization is rather conservative because efficient serpentinization can also occur at higher temperatures and pressures (*19*).

In a second step, we refine the assessment of serpentinization and natural H_2_ generation potential in our modelled rift-inversion orogens by following the methodology from (*12*) that was previously used for rift settings (Fig. 1C). This methodology involves the tracing of large active mantle fault zones that are assumed to serve as primary conduits for the water circulation required for serpentinization of mantle rocks (Fig. 1C). We subsequently apply the temperature-dependent bell curve formula (*12*) to calculate the serpentinization capacity (kilogram of serpentinized mantle per year) along these faults, which is defined as per kilometer perpendicular to the model (i.e., along-strike of the rift or rift-inversion orogen in question) (Fig. 3, D to F). Last, we derive the natural H_2_ capacity (natural H_2_ generation per year) that is defined as mole per kilometer perpendicular to the model by assuming that 1 kg of serpentinized mantle may generate between 100 and 300 mmol of natural H_2_ (Fig. 3, D to F) (*11*, *23*).

Our approach provides a holistic but simplified first-order assessment of natural H_2_ potential in rift-inversion orogens; in the Material and Methods section, we present extensive details on model design and important nuances to our model analysis [e.g., the 2D nature of our models, the focus on serpentinization of exhumed mantle material within the 200° to 350°C temperature window and the use of the temperature-determined serpentinization formula from Liu *et al.* (*12*) for our analysis, the assumption that the water needed for serpentinization is readily available in active fault zones, and the impact of mantle rock composition and water chemistry on natural H_2_ generation]. In the supplementary data (*37*), an overview of all model results produced for this study is presented, along with results of additional parameter tests demonstrating the robustness of our results regarding serpentinization parametrization, and of our assessment of natural H_2_ potential during rift-inversion orogen evolution.

### Impact of limited rifting: Model A

Model A represents the impact of a short initial rifting duration (Figs. 2, A and B and 3, A and D). During the initial 5 million years (Myr) of rifting, a symmetric fault-bounded basin form (Fig. 2A). Once rifting ends, shortening ensues right away (Fig. 2B). The rift basin is inverted within 5 Myr, and a largely symmetric orogen consisting of crustal material with a small core of mantle material develops (Fig. 2B). The short rifting phase allows for a small linear increase in exhumed mantle area (until a maximum of ~1 × 10^9^ m^2^), but almost all mantle material is buried again during subsequent inversion (Figs. 2, A and B and 3A). Consequently, very little mantle material reaches the adopted serpentinization window, and an absence of large faults means very limited water circulation (Fig. 2, A and B). Hence, the serpentinization capacity remains below 1 × 10^8^ kg year^−1^ per along-strike kilometer (Fig. 3, A and D).

### Impact of longer rifting and post-rift cooling: Models B and C

Models B and C reveal the impact of longer rift durations and a post-rift cooling phase (Figs. 2, C to J and 3, B and C and E and F). After 15 Myr of rifting in model B, mantle material is exhumed near the surface with only a sparse (1 to 4 km) cover of crustal material and sediments (Fig. 2, C and F). The 25 Myr of rifting in model C leads to break up, with only 1 to 4 km of sediment cover remaining (Fig. 2G). Mantle exhumation over time follows a similar linear increase as in model A but is much more prominent (~3.5 × 10^9^ m^2^ and ~7 × 10^9^ m^2^ at the end of rifting in models B and C, respectively Fig. 3, B and C). In both models, the actively exhuming mantle area deviates from the total exhumed mantle trend after 8 Myr (Fig. 3, B and C). The total area of exhumed mantle remains stable during the subsequent 20 Myr of post-rift cooling, apart from a slow gradual decline due to thermal sag and sediment accumulation (Fig. 3, B and C). However, a large part of exhumed mantle material continues to rise due to isostatic balancing of rift shoulder erosion (Fig. 3, B and C). During inversion, most exhumed mantle is buried again, but the formation of an asymmetric orogen causes a wedge of fresh mantle material to be thrust on top of the down-going plate, near the surface, and well within the adopted serpentinization window (Figs. 2, D and E and H to J and 3, B and C).

Large amounts of mantle material are found in the adopted serpentinization window by the end of rifting in models B and C, which further increases during post-rift cooling (Figs. 2F and 3, B and C). Consequently, serpentinization and natural H_2_ generation capacities at the end of rifting in models B and C are much higher than in model A (Fig. 3, D to F). However, the maximum serpentinization capacity of ~5 × 10^9^ kg(km year)^−1^ and the associated maximum natural H_2_ generation capacity of ~1.5 × 10^9^ mol(km year)^−1^ during rifting are similar in both models, suggesting an upper limit imposed by the vertical extent of the adopted serpentinization window (Fig. 3, E and F). Serpentinization drops to zero during post-rift cooling as no strain localizes (and thus no water is expected to circulate in our analysis; see the Materials and Methods) in the serpentinization window (Figs. 2F and 3, E and F).

**Fig. 4.**
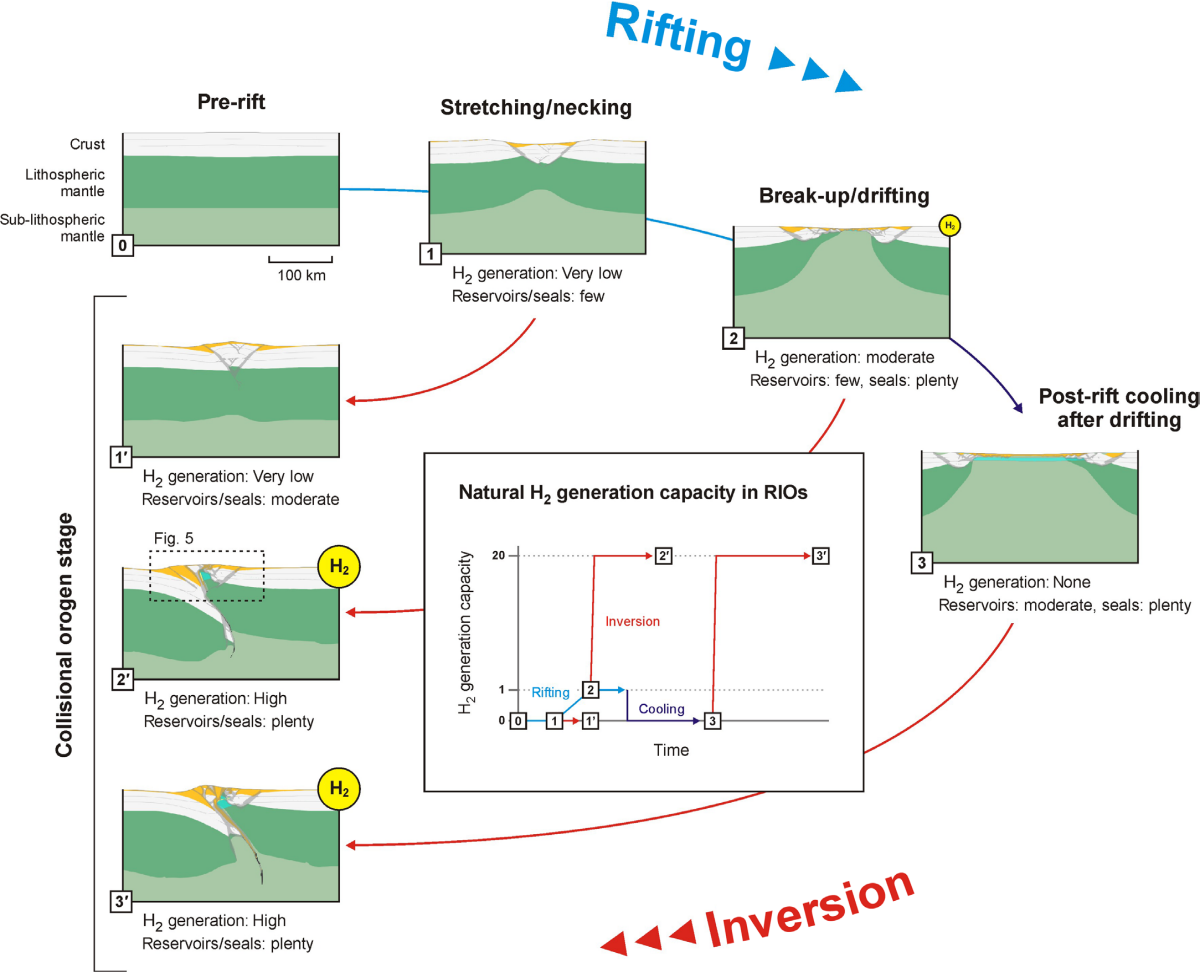
Schematic summary of the evolution of natural H_2_ potential during rifting and subsequent inversion, depending on rift duration and post-rift cooling. Natural H_2_ capacity (H_2_ generation per time unit, as indicated by the yellow bubbles in the sections, as well as in the central graph) occurs in the more advanced rifting stages but can be up to 20 times higher during inversion. The assumption that large-scale faulting and associated water circulation is required for serpentinization to occur is highlighted by the absence of natural H_2_ generation during post-rift cooling in our analysis. We also specify the likelihood of reservoir (e.g., sands) and seal (e.g., clays) being available to allow for natural H_2_ accumulation during the various stages of rift-inversion orogen evolution. Light green: Sub-lithospheric mantle; dark green: lithospheric mantle; white: crust; orange: sediments; turquoise: mantle material in the adopted serpentinization window (between the 200° and 350°C isotherms); transparent gray: fault zones; black: active fault zones in mantle; yellow: areas of highly efficient serpentinization (the H_2_ kitchen). See also Fig. 1C for legend. RIO, rift-inversion orogen.

The subsequent overthrusting of fresh mantle material within a relatively cool orogen focuses strain along mantle fault zones within the adopted serpentinization window, but on a much larger scale than during previous rifting (Fig. 2, C to J). From the start of inversion, serpentinization and natural H_2_ generation capacity in both models B and C are around 1 × 10^11^ kg(km year)^−1^ and 3 × 10^10^ mol(km year)^−1^, respectively, about 20 times higher than at the end of rifting (Fig. 3, E and F). This trend is only interrupted by the notable drop in serpentinization capacity at *t* ≈ 50 Myr in model B indicating a temporary strain migration away from the serpentinization window (Fig. 3E). These values [also found in other models with sufficient initial rifting; see (*37*)] indicate that serpentinization and H_2_ generation capacity during inversion can be up to 20 times higher than during rifting but may similarly have an upper limit imposed by the vertical extent of the adopted serpentinization window (Figs. 3, E and F and 4).

## DISCUSSION

### Mantle exhumation patterns and natural H_2_ potential

Our numerical models show that rift duration is the key factor enabling exhumation and preservation of mantle material in rift-inversion orogens (Figs. 2 to 4). Sufficient rifting ensures that mantle material is exhumed to begin with, but rifting also facilitates the formation of asymmetric orogens in which mantle rocks may be preserved or further exhumed [as qualitatively observed in previous numerical modeling studies, e.g., (*27*, *30*–*32*)].

Given sufficient time, rifting can exhume large mantle volumes (Figs. 1 to 4). However, the high syn-rift thermal gradients limit the size of the serpentinization window and serpentinization capacity during rifting [up to ~5 × 10^9^ kg(km year)^−1^, similar to values calculated for rifting in other studies (*10*, *12*)]. By contrast, our analysis shows that the maximum serpentinization capacity during inversion can be about 20 times higher [~1 × 10^11^ kg(km year)^−1^] due to the much larger serpentinization window in a colder orogenic setting and larger fault zones enabling ample water circulation (Figs. 2 to 4). Still, without active deformation, such as during post-rift cooling, water circulation may be reduced or halted, so that serpentinization is limited or stops altogether (Figs. 2F; 3, E and F; and 4). This need for water circulation along (active) faults for mantle rocks to serpentinize can explain the occurrence of relatively fresh mantle outcrops in orogens [e.g., in the Pyrenees (*38*, *39*)], since our models show that mantle material may exhume without being near these large faults.

The serpentinization capacity in our models is convertible to potential natural H_2_ generation capacity, which we estimate to be at most ~1.5 × 10^9^ mol(km year)^−1^ during advanced rifting and up to 20 times higher [~3 × 10^10^ mol(km year)^−1^] during inversion (Figs. 3 and 4). Furthermore, the resulting rift-inversion orogens generally contain large sediment volumes that can act as reservoirs and seals when natural H_2_ is generated in bulk during inversion (*21*). For example, reservoirs may consist of permeable coarse-grained deposits (sands and gravel) formed in proximal environments throughout both rifting and inversion, whereas seals may consist of impermeable fine-grained deposits (clay) that originally formed in distal rift settings before being incorporated in the orogen. In contrast, reservoir-quality sediments are most likely absent in the distal rift environments dominated by fine-grained deposits at the time of bulk H_2_ generation during advanced rifting (Figs. 1 to 4).

**Fig. 5.**
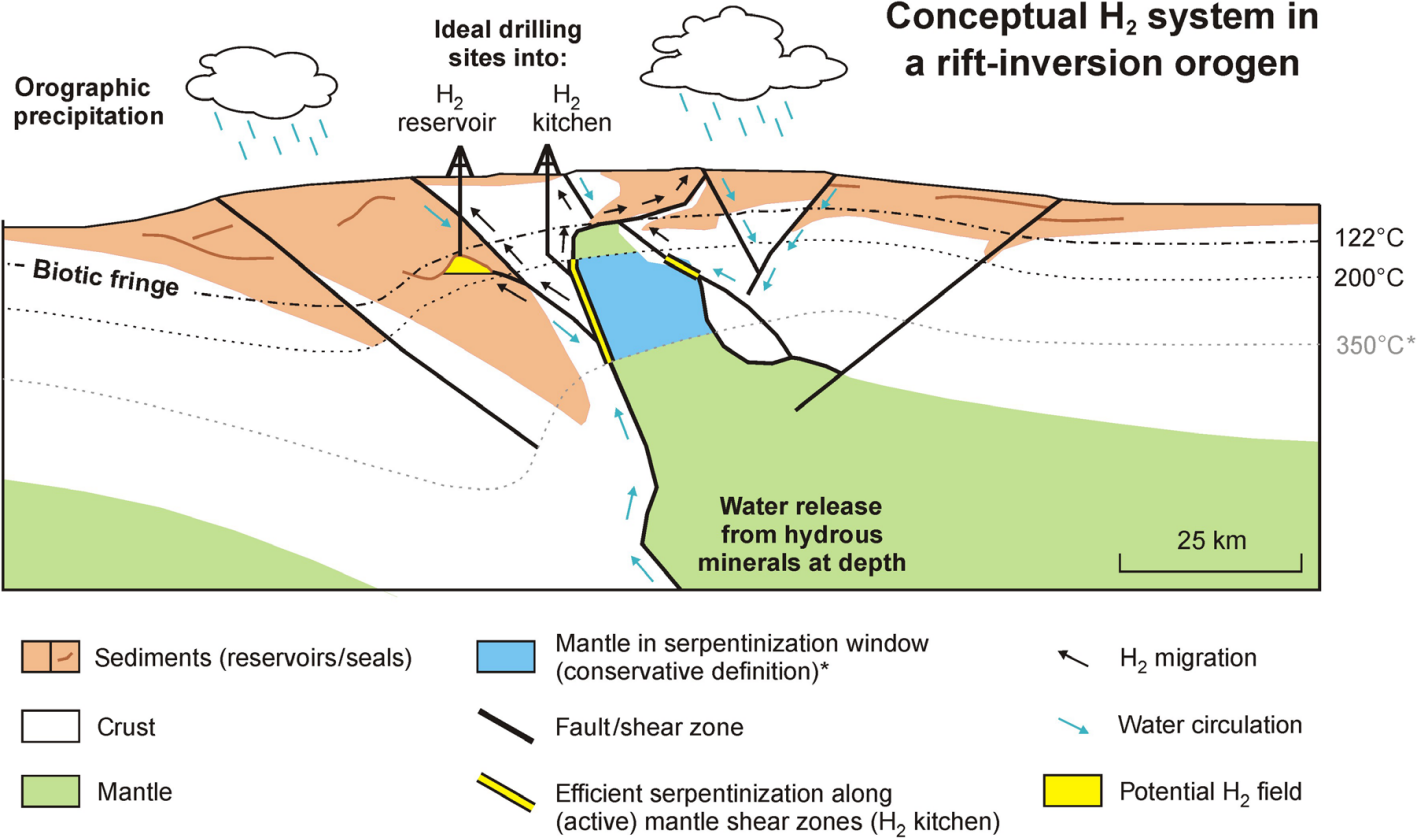
Conceptual depiction of a natural H_2_ system linked to serpentinization of exhumed mantle in the overriding plate of a rift-inversion orogen. Figure based on the geology of panel (2′) from Fig. 4. Water, originating from either the deep or the surface, can flow along (active) fault zones toward those parts of the mantle that are in the (*conservatively defined, see main text for details) serpentinization window (200° to 350°C), where natural H_2_ generation is considered most efficient (the H_2_ kitchen). The H_2_ gas can subsequently migrate toward the surface along fault zones and may accumulate in reservoirs capped by seals of impermeable layers within sedimentary units. Ideally, this natural H_2_ accumulation occurs below the “biotic fringe” (the 122°C isotherm that indicates the limit of life) above which H_2_ is not consumed by microbial activity (*20*, *40*), so that a conventional natural H_2_ field may develop that can be exploited. Alternatively, it would be possible to directly drill into the natural H_2_ kitchen and stimulate the mantle source rock to enhance natural H_2_ generation (*41*).

These reservoirs and seals are crucial for capturing natural H_2_ migrating from the serpentinizing mantle (*24*) and would enable the establishment of a fully fledged orogenic “hydrogen system,” a concept analogous to “petroleum systems” (*33*). Such a hydrogen system involves an exhumed mantle source rock in the serpentinization window generating natural H_2_ (the “H_2_ kitchen”) and its subsequent migration to reservoirs with a structure and seal, where the natural H_2_ can accumulate for drilling and extraction (Fig. 5) (*21*). The orogenic hydrogen system concept we propose here is conservative, as it focuses on bulk natural H_2_ generation by a H_2_ kitchen situated in the exhumed mantle wedge, which we believe provides the best chance for accumulation of economical volumes to form exploitable H_2_ fields. However, additional potential, not considered in this study, may lie in contributions from efficient serpentinization and natural H_2_ generation at higher temperatures and pressures (*19*) or natural H_2_ generated by less efficient serpentinization elsewhere in the system that may accumulate in considerable volumes over geological time.

Whatever the source of the natural H_2_, its preservation during migration and after arrival in a reservoir is crucial for any meaningful accumulation to form. Hence, natural H_2_ fields should ideally have reservoir temperatures above the “biotic fringe” (the ~122°C isotherm that indicates the limit of life) above which H_2_ is not consumed by microbial activity (*20*, *40*) (Fig. 5). Alternatively, when no H_2_ fields are available, the serpentinizing mantle rocks in the H_2_ kitchen themselves could be directly targeted and stimulated (*41*) for natural H_2_ extraction (Fig. 5). This natural H_2_ extraction could be combined with other resource exploitation efforts as well (*41*) (see Materials and Methods).

The key to successful natural H_2_ exploration will be the application of a “natural hydrogen system analysis” approach that assesses the spatial and temporal aspects of a natural H_2_ system, similar to the “petroleum system analysis” approach commonly used for hydrocarbon exploration (*6*, *21*, *33*, *42*).

### Promising natural H_2_ exploration sites in the Alpine-Himalayan orogenic belt

**Fig. 6.**
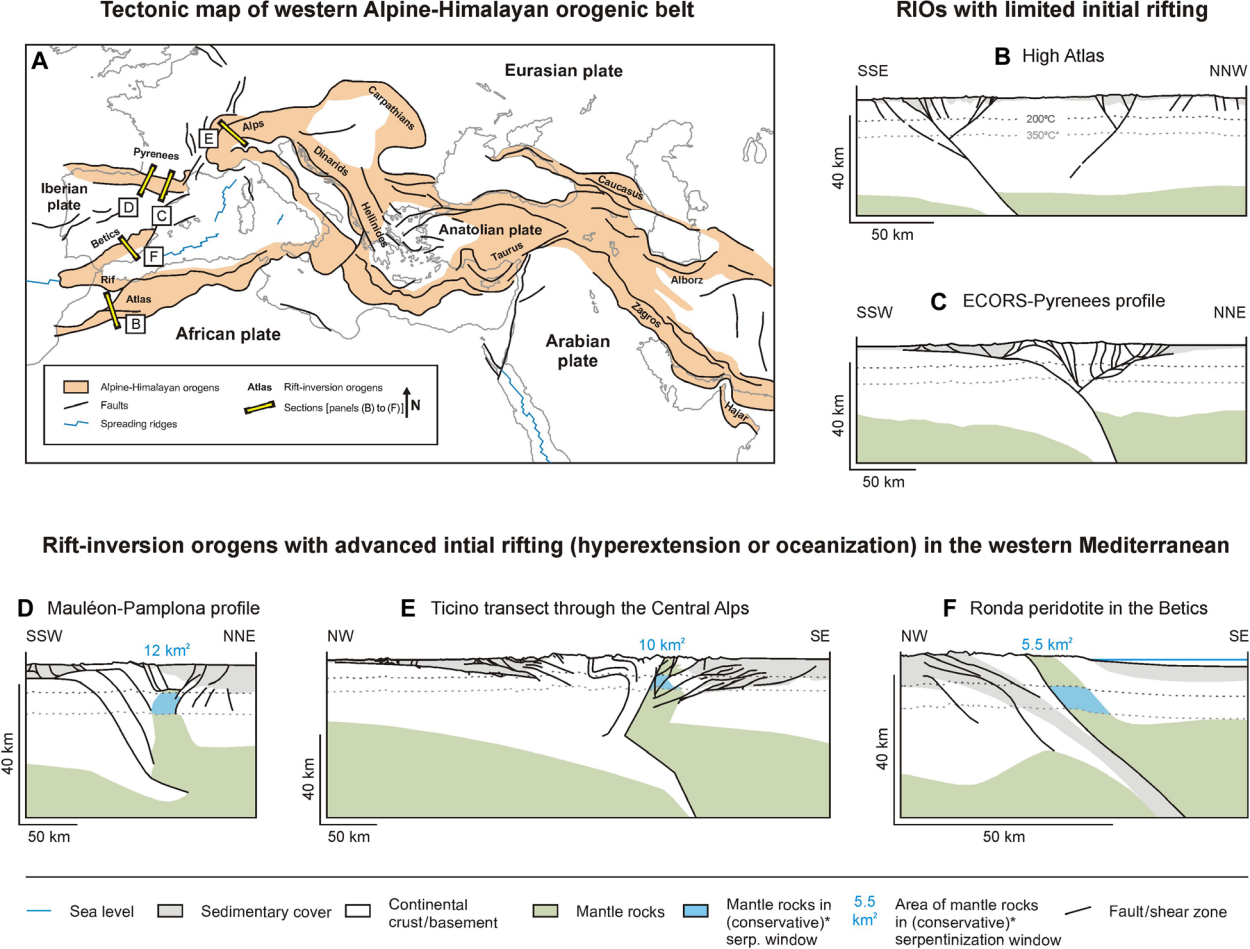
Examples of rift-inversion orogens (RIOs) in the western part of the Alpine-Himalayan orogenic belt. (**A**) Tectonic map of the western Alpine-Himalayan orogenic belt, showing the locations of the sections in (**B**) to (**F**), each of which contains a first-order estimation of the in-section area of exhumed mantle material found in the (*conservatively defined, see text for details) serpentinization window (200° to 350°C), assuming a standard geotherm of 25°C km^−1^ (*75*). Modified and simplified after Woudloper (*76*) (used under a CC BY-SA 1.0 license; https://creativecommons.org/licenses/by-sa/1.0/) and Faccenna *et al.* (*77*). [(B) and (C)] High Atlas and Eastern Pyrenees: Rift-inversion orogen with little natural H_2_ potential, as far as serpentinization of mantel material is concerned because no mantle is found in or near the serpentinization window. Modified and simplified after Muñoz (*78*), with permission of the Geological Society, London, and Arboleya *et al.* (*79*). [(D) to (F)] Western Pyrenees, Central Alps, and Betics: Rift-inversion orogens with advanced initial rifting that contain exhumed mantle material within the serpentinization window (i.e., the Mauléon body, Ivrea mantle wedge, and Ronda peridotite massif, respectively). Modified and simplified after Mazzoli *et al.* (*50*), Rosenberg and Kissling (*80*) (compilation based on sources cited therein), and Wang *et al.* (*44*), used with permission of the Geological Society, London, and of the Geological Society of America.

Our modeling results enable a solid, first-order evaluation of the natural H_2_ potential associated with exhumed mantle as part of the overriding plate in rift-inversion orogens. A key result of our study is that relatively cold rift-inversion orogens, such as those in the western Alpine-Himalayan orogenic belt (Fig. 6), provide much better environments for natural H_2_ exploration due to their much larger serpentinization window than is the case in relatively hot rift, rifted margin, and mid-oceanic ridge settings.

The Pyrenees, which are characterized by a gradient in initial rift basin maturity from limited rifting in the east to hyperextension in the west, represent a potential target area for natural H_2_ exploration (*29*, *43*) (Fig. 6, C and D). While no evidence for exhumed mantle exists in the eastern Pyrenees (Fig. 6C) (*29*, *43*), its presence underneath the Mauléon Basin to the west is well established (Fig. 6D) (*44*–*46*). Recent studies in this inverted basin showed that natural H_2_ generation may be on-going here, compatible with the fact that the well-identified exhumed mantle body is in the serpentinization window (*21*, *25*, *26*) (Fig. 6D). Given the conservative maximum natural H_2_ generation capacity value of ~3 × 10^10^ mol(km year)^−1^, we obtain for rift-inversion orogens, and assuming a moderate 10 km extent of the exhumed mantle body along-strike, up to 3 × 10^11^ mol (i.e., 6 × 10^8^ kg) of natural H_2_ could be generated below the Mauléon Basin each year. Adopting an average yearly primary energy use of 3.6 × 10^4^ kWh per person in France (*47*), the caloric value of such a quantity of natural H_2_ derived from this mantle body alone (~2 × 10^11^ kWh) would cover the yearly energy needs of the nearby city of Toulouse and its population of ~500,000 inhabitants. Together with the presence of thick sedimentary deposits, including extensive evaporite layers that form ideal seals, all ingredients may be in place for a functional hydrogen system where natural H_2_ can accumulate to volumes considerably larger than the yearly generation rate (Figs. 5 and 6D) (*21*).

The wider western Alpine-Himalayan orogenic belt contains various rift-inversion orogens that formed after the closure of a number of small oceanic basins [e.g., (*28*, *48*, *49*) (Fig. 6, A, E and F)]. For instance, the Southern Alps contain exhumed mantle material (Ivrea mantle wedge) situated at suitable depths in the retro-wedge (Fig. 6E) (*28*), which, in combination with indications of fossil natural H_2_ generation found in exposed mantle rocks (*22*–*24*), makes for a promising natural H_2_ exploration target area. Also, the Betics (Ronda peridotite massif) and the Balkans (remnants of the Meliata-Vardar ocean) contain various exhumed mantle bodies that are linked to abiotic gas generation and warrant further exploration (Fig. 6, A and F) (*16*, *17*, *48*, *50*, *51*). On a larger scale, the branches of the Alpine-Himalayan orogenic belt stretching farther eastward into Asia, where various mantle bodies that are exhumed could be of great interest for natural H_2_ exploration as well (Fig. 6A) (*52*).

### Implications and future perspectives

New geo-resources are urgently needed to facilitate the energy transition. To address this need, it is crucial to develop novel concepts and exploration strategies rooted in research-driven approaches. One such concept is the extraction of natural H_2_ generated by serpentinization of exhumed mantle material in rift-inversion orogens. Its potential is highlighted by both existing field data [e.g., (*16*, *17*)], and our new modelling results. The current state of natural H_2_ exploration may be analogous to that of petroleum exploration before the 1859 Drake oil discovery in Pennsylvania, which triggered the rise of the oil industry (*6*). Future work is needed to develop robust strategies for natural H_2_ exploration in rift-inversion orogens, especially given the additional economic opportunities linked to natural H_2_ exploitation, such as production of associated CH_4_, geothermal energy generation, CO_2_ storage, and mineral extraction (*23*, *41*).

These exploration strategies may be informed by our modelling results, which allow the quantification of the mechanisms and timing of tectonic processes that facilitate large-scale serpentinization and natural H_2_ generation during rift-inversion orogen evolution (Figs. 2 to 4). Moreover, our modeling work provides first-order insights into the dynamic spatial and temporal links between the location of natural H_2_ generation (source/H_2_ kitchen), potential natural H_2_ migration pathways (faults), and reservoirs (sediments), where natural H_2_ may accumulate in orogenic hydrogen systems (Fig. 5). Similar to exploration efforts in petroleum systems, understanding the dynamic evolution of these links will be critical for accurately characterizing hydrogen systems and identifying viable targets for natural H_2_ exploitation as well as associated economic resource extraction opportunities.

## MATERIALS AND METHODS

### Numerical method

**Fig. 7.**
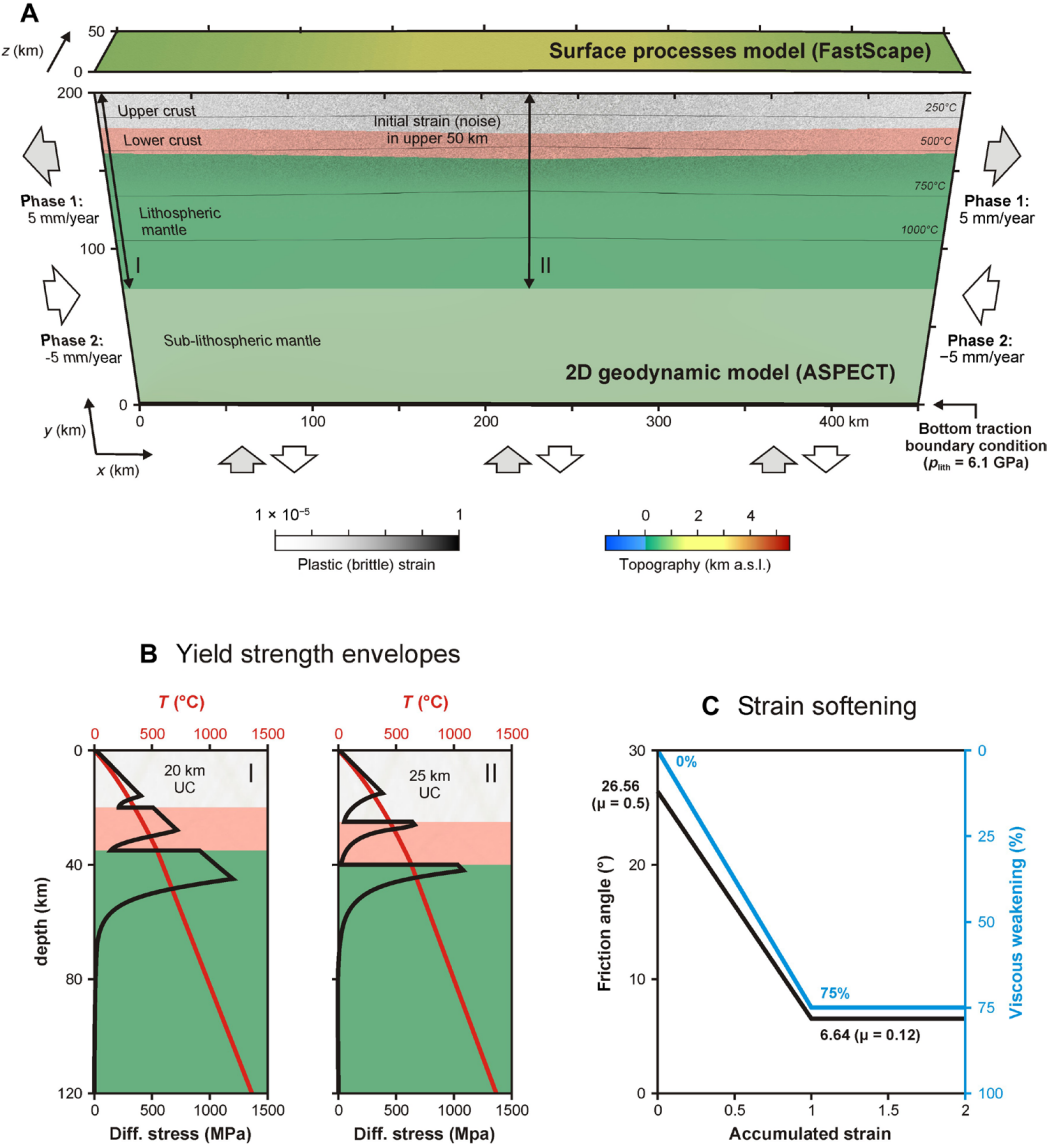
Reference model setup. (**A**) 3D visualization of the 2D geodynamic model (ASPECT) with the quasi-3D surface processes model (FastScape) on top. Black lines indicate temperature contours, and initial strain (noise) at *t* = 0 Myr is present in the uppermost 50 km. (I) and (II) indicate the outer and central locations of the yield strength profiles depicted in (B), which have different crustal thicknesses as the upper crust in the model center is 25 km thick, instead of the 20 km at the model edge. (**B**) Yield strength and temperature profiles along (I) and (II) shown in (A). (**C**) Plastic (brittle) and viscous weakening intervals applied in our models. Modified after Neuharth *et al.* (*36*). UC, upper crust.

We base our modelling approach on previous work by Neuharth *et al.* (*36*) using the thermo-mechanical geodynamic code ASPECT (Advanced Solver for Planetary Evolution, Convection, and Tectonics) (*34*, *35*, *53*–*57*) coupled with FastScape (*36*, *58*–*60*) for the inclusion of surface processes (Figs. 1 and 7). We apply a modified version of ASPECT 2.4.0-pre for the coupling with FastScape. ASPECT and FastScape source code and installation details, the custom ASPECT plugins we use, the parameter files used for each model run, the log files of each model run, and the ParaView state files used for model analysis are provided in a publicly accessible Zenodo repository (*61*) (https://doi.org/10.5281/zenodo.14672886).

#### Geodynamic modeling (ASPECT)

We apply ASPECT to solve the conversation equation under the extended Boussinesq approximation.

Conservation of momentum is described by the following equation

$$-\nabla \cdot 2\eta \overset{\cdot }{\varepsilon }\left(u\right)+\nabla P=\rho g$$ (1)

where η is viscosity, $\overset{\cdot }{\varepsilon }$ is the deviatoric strain rate, ***u*** is velocity, *P* is pressure, ρ is density, and g is gravitational acceleration.

Conservation of mass is described as follows

∇·u=0 (2)

Conservation of energy is defined as

$$\bar{\rho }C_{p}\left(\frac{\partial T}{\partial t}+u\cdot \nabla T\right)-\nabla \cdot k\nabla T=\bar{\rho }H+\left(2\eta \overset{\cdot }{\varepsilon }\right):\overset{\cdot }{\varepsilon }+\alpha \rho T\left(u\cdot g\right)$$ (3)

where $\bar{\rho }$ is the reference adiabatic density, *C*_p_ the specific heat capacity, *T* the temperature, *k* the thermal conductivity, *H* the radiogenic heating, and α the thermal expansivity. The right-hand terms represent radioactive heating, viscous shear heating, and adiabatic heating, respectively. See Table 1 for the used parameter values.

**Table 1.** ASPECT parameters.

Parameter	Symbol	Units	Sediment	Upper crust	Lower crust	Lithospheric mantle	Sub-lithospheric mantle
Reference surface density*	⍴_0_	kg m^−3^	2520	2700	2850	3280	3300
Adiabatic surface temperature	*T*_AS_	°C	1284	1284	1284	1284	1284
LAB isotherm temperature	*T*_LAB_	°C	1284	1284	1284	1284	1284
Thermal expansivity	α	K^−1^	3.7 × 10^−5^	2.7 × 10^−5^	2.7 × 10^−5^	3.0 × 10^−5^	3.0 × 10^−5^
Thermal diffusivity	κ	m^2^ s^−1^	7.28 × 10^−7^	7.72 × 10^−7^	7.31 × 10^−7^	8.38 × 10^−7^	8.33 × 10^−7^
Heat capacity	*C*_p_	J kg^−1^ K^−1^	1200	1200	1200	1200	1200
Heat production	*H*	W m^−3^	1.2 × 10^−6^	1.0 × 10^−6^	0.1 × 10^−6^	0	0
Cohesion	*C*	Pa	5 × 10^6^	5 × 10^6^	5 × 10^6^	5 × 10^6^	5 × 10^6^
Internal friction angle (unweakened)	ɸ	°	26.56	26.56	26.56	26.56	26.56
Plastic strain weakening interval	–	–	[0,1]	[0,1]	[0,1]	[0,1]	[0,1]
Plastic strain weakening factor	ɸ*_wf_*	–	0.25	0.25	0.25	0.25	0.25
Viscous strain weakening interval	–	–	[0,1]	[0,1]	[0,1]	[0,1]	[0,1]
Viscous strain weakening factor	–	–	0.25	0.25	0.25	0.25	1.0
Creep properties^†^			Wet quartzite	Wet quartzite	Wet anorthite	Dry olivine	Wet olivine
Stress exponent (dis)	*n*	–	4.0	4.0	3.0	3.5	3.5
Constant prefactor (dis)	*A*_dis_	Pa^-*n*^s^−1^	8.57 × 10^−28^	8.57 × 10^−28^	7.13 × 10^−18^	6.52 × 10^−16^	2.12 × 10^−15^
Activation energy (dis)	*E*_dis_	J mol^−1^	223 × 10^3^	223 × 10^3^	345 × 10^3^	530 × 10^3^	480 × 10^3^
Activation volume (dis)	*V*_dis_	m^3^ mol^−1^	0	0	38 × 10^−6^	18 × 10^−6^	11 × 10^−6^
Constant prefactor (diff)	*A*_diff_	Pa^−*1*^ s^−1^	5.79 × 10^−19^	5.79 × 10^−19^	2.99 × 10^−25^	2.25 × 10^−9^	1.5 × 10^−9^
Activation energy (diff)	*E*_diff_	J mol^−1^	223 × 10^3^	223 × 10^3^	159 × 10^3^	375 × 10^3^	335 × 10^3^
Activation volume (diff)	*V*_diff_	m^3^ mol^−1^	0	0	38 × 10^−6^	6 × 10^−6^	4 × 10^−6^
Grain size (diff)	*d*	m	0.001	0.001	0.001	0.001	0.001
Grain size exponent (diff)	*m*	–	2.0	2.0	3.0	0	0

*Model input densities are scaled so that at surface temperatures (*T*_0_ = 273 K or 0°C) these values are reached.

†Creep properties: dis = dislocation creep, diff = diffusion creep.

Each compositional field *c*_i_ (e.g., representing lithologies such as the upper crust or strain fields) is advected with the calculated velocity field

$$\frac{\partial c_{i}}{\partial t}+u\cdot \nabla c_{i}=q_{i}$$ (4)

where reaction rate *q*_i_ is nonzero for the plastic (brittle) and viscous strain fields.

#### Landscape evolution modeling (FastScape)

FastScape modifies ASPECT’s model domain’s surface as a function of stream-power law fluvial erosion, hillslope and marine diffusion, horizontal advection, and vertical uplift (where in the latter two cases, the *X* and *Y* velocities and the *Z* velocities from ASPECT are used as model input) (*58*–*60*). Topography changes in the continental domain ($h\geq h_{\text{sea}}$) are therefore described by the following equation

$$\frac{\mathrm{dh}}{\mathrm{dt}}=U-K_{f}A^{m}S^{n}+\frac{G}{A}\int_{A}\left(U-\frac{\mathrm{dh}}{\mathrm{dt}}\right)\mathrm{dA}+K_{c}\nabla^{2}h+v\cdot \nabla h$$ (5)

where *h* is the topographic elevation, *U* the uplift rate, *K*_f_ the bedrock erodibility, *A* the drainage area, *S* the slope, *m* the drainage area exponent, *n* the slope exponent, *G* the deposition coefficient, *K*_c_ the continental diffusion coefficient, and ***v*** the horizontal velocity. See Table 2 for the used parameter values.

**Table 2.** FastScape parameters.

Parameter	Symbol	Unit	Value
Drainage area exponent	*m*	–	0.4
Slope exponent	*n*	–	1
Bedrock/sediment diffusivity	*K*_c_	m^2^ year^−1^	5 × 10^−3^
Bedrock/sediment erodibility	*K*_f_	m^0.2^ year^−1^	1 × 10^−5^
Bedrock/sediment deposition coefficient	*G*	–	1
Marine diffusivity	*K*_m_	m^2^ year^−1^	200
Sand/shale ratio	*F*	–	1
Sand/shale porosity	φ	–	0
Sand/shale e-folding depth	*z*	m	0
Depth averaging thickness	*L*	m	100
Background sedimentation rate	*Q*_0_	m year^−1^	0

Moreover, marine processes (*h* < *h*_sea_) are described as follows

$$\frac{\mathrm{dh}}{\mathrm{dt}}=K_{m}\nabla^{2}h+Q_{s}+v\cdot \nabla h+Q_{o}$$ (6)

where *K*_m_ represents the marine diffusion coefficient, *Q*_s_ the sediment flux from the continent to the marine domain at the continent-marine boundary (*60*), and *Q*_o_ a homogeneous marine background sedimentation rate.

### Geodynamic model design

#### Model geometry

Our 2D rectangular domain is 450-km long and 200-km high and contains three layers together representing a 115-km-thick stable continental lithosphere: (i) a 20-km-thick wet quartzite upper crust (*62*), (ii) a 15-km-thick wet anorthite lower crust (*63*), and (iii) an 80-km-thick lithospheric mantle consisting of dry olivine (*64*, *65*), overlying a wet olivine asthenosphere (*66*) (Fig. 7A and Table 1). Thicker upper crust (25 km) in the model domain center (between *x* = 150 km and *x* = 300 km, following a Gaussian distribution) produces a weaker strength profile and localizes kinematically driven tectonic deformation in this region (Fig. 7B). Furthermore, a randomized initial plastic (brittle) strain pattern with a maximum value of 0.5 is introduced in the upper 50 km of the crust to assist strain localization (Fig. 7A). The initial temperature follows a 1D steady-state continental geotherm in the lithosphere (*67*) and an adiabatic profile below.

#### Viscoplastic rheology and weakening processes

We use a visco-plastic rheology (*68*) that combines diffusion creep, dislocation creep and Drucker-Prager plasticity. The friction angle is linearly weakened up to four times between brittle strain values of 0 and 1 (Fig. 7C and Table 1). Similarly, we apply a four times pre-yield viscous weakening between these same values of the viscous strain. Picard iterations on the Stokes solution are used to solve up to a nonlinear tolerance of 2 × 10^−5^ for a maximum of 120 iterations per time step.

#### Boundary conditions

A fixed temperature of 0°C is prescribed at the surface and a temperature of 1361°C at the bottom boundary. Tectonic deformation (divergence and convergence) is induced by prescribing outward and inward flow on the lateral model boundaries, respectively (Fig. 7A). This prescribed horizontal boundary velocity is 5 mm year^−1^ on either lateral boundary, making for a total velocity of 10 mm year^−1^ during either rifting or inversion. Flow through the lateral boundaries is compensated by inflow and outflow through the bottom boundary, which is controlled by a traction boundary condition set to a lithostatic pressure of 6.1 × 10^9^ Pa (Fig. 7A). The latter boundary condition is imposed to conserve mass within the ASPECT model over time and to ensure isostatically balanced topography. Tectonic quiescence (leading to post-rift cooling) between divergence and convergence phases is simulated by prescribing a velocity of zero at the lateral model boundaries.

#### FastScape parameters

We adopt a sea-level of 500 m below the initial surface level of the ASPECT model domain, a value that corresponds to the average elevation of stable continents with ~35-km crust far away from plume-related hotspot swells (*69*). All FastScape parameters are specified in Table 2. In particular, we apply a fluvial erosion coefficient (*K*_f_) of 10^−5^ m^0.2^ year^−1^ and a continental hillslope diffusivity coefficient (*K*_c_) of 5 × 10^−3^ m^2^ year^−1^ that are in agreement with previous model and observational constraints [see (*59*) and references therein].

#### Discretization

The ASPECT mesh cell size is 10 km at the bottom of the domain, with a three-step refinement down to 1250 m above the 750°C isotherm, to properly capture brittle deformation in the colder parts of the lithosphere. We use second-order (Q2Q1) elements, and to optimally visualize the solution, results are output and analyzed on a grid with double the resolution of each cell (Fig. 7), the maximum resolution being 625 m.

### Varied model parameters

We systematically test the impact of plate tectonic parameters in our rift-inversion orogen models (Table 3). First, we apply different initial rifting durations of 5, 15, and 25 Myr, at a total plate motion velocity of 10 mm year^−1^. These values roughly reflect the varying degrees of rifting before convergence [e.g., in the High Atlas, Pyrenees, and Alps, e.g., (*22*, *28*, *70*)], while the total convergence duration in all models is equal to the initial rifting duration plus 5 Myr to allow for initial collision to occur. Second, we apply a post-rift tectonic quiescence period of 0 or 20 Myr, which also reflect natural rift-inversion orogen settings (*22*, *28*, *70*).

**Table 3.** Variable model parameters.

Model name (main text)	Model name [Materials and Methods section and supplementary data (*37*, *61*)]	Rifting duration	Post-rift cooling duration	Inversion duration	Total model duration
**A**	**M1**	5 Myr	0 Myr	10 Myr	15 Myr
–	M2	15 Myr	0 Myr	20 Myr	35 Myr
–	M3	25 Myr	0 Myr	30 Myr	55 Myr
–	M4	5 Myr	20 Myr	10 Myr	35 Myr
**B**	**M5**	15 Myr	20 Myr	20 Myr	55 Myr
**C**	**M6**	25 Myr	20 Myr	30 Myr	75 Myr

### Model analysis

Using the open source ParaView visualization software (www.paraview.org), we extract for each model the following three overall metrics, for each model data output interval of 500 kyr: the area of exhumed mantle (i.e., the total area of mantle above the initial Moho at 35 km depth; Figs. 1 and 7), the area of actively rising exhumed mantle (i.e., the area of exhumed mantle with upward vertical velocity > 0 m s^−1^), and the area of mantle material within the adopted serpentinization window (i.e., the 200° to 350°C temperature window, in which serpentinization is considered to be most efficient if the water required for the reaction would be available).

Note that this serpentinization window extent is rather conservative as efficient serpentinization can also occur at higher temperatures and pressures, particularly during inversion and subduction [e.g., (*19*, *23*)], but we base our analysis of natural H_2_ potential on the temperature-dependent serpentinization formula from Liu *et al.* (*12*, *18*). According to this formula, peak serpentinization efficiency (and thus bulk serpentinization) follows a bell curve that is centered in the 200° to 350°C serpentinization window, with very minor serpentinization occurring outside this window (Fig. 1C). This serpentinization window is based on conditions in rift systems (*12*). Although efficient serpentinization may also occur at higher pressures and temperatures [e.g., (*19*, *23*)], we applied the same equation for assessing natural H_2_ potential throughout the evolution of our modeled rift-inversion orogens to ensure an internally consistent analysis. We believe that this is permissible because tests with a larger serpentinization window (200° to 500°C) indicate that the main insights from our analysis remain robust (for more details, see also the “Nuance to our model analysis” section on nuances to our approach).

We subsequently adopt the general approach of Liu *et al.* (*12*) to calculate serpentinization capacity in kg(km year)^−1^ and associated H_2_ generation potential in mol(km year)^−1^ in geodynamic models (Fig. 1C). This approach is based on the first-order assumption that active and sufficiently mature faults feature a high permeability, which allows for ample water circulation to enable high-degree serpentinization of mantle rocks at any moment in time. Therefore, at each output time step, we select the model mesh cells with mantle rocks that have accumulated a total plastic (i.e., brittle) strain ε ≥ 0.5 and that are undergoing considerable active deformation (second strain rate invariant $\overset{\cdot }{\varepsilon }$ ≥ 2.5 × 10^−15^). Note that we also test different strain and strain rate thresholds (ε ≥ 0.1 or 1, and $\overset{\cdot }{\varepsilon }$ = ≥ 1.5 × 10^−15^ or 5 × 10^−15^) to estimate the variability in the results of our analysis [see (*37*)]. We find that these reasonably different thresholds do not meaninfully change our conclusions.

Taking the selected model mesh cells with actively deforming mantle rocks, we apply the temperature-dependent serpentinization reaction formulas from (*12*, *18*) to compute serpentinization rates in these mesh cells using ParaView’s Python calculator application

$$\frac{\partial \text{Dserp}}{\partial t}={\mathrm{Ae}}^{\left[-b_{s}{\left(T-c_{s}\right)}^{2}\right]}$$ (7)

where $\frac{\partial \text{Dserp}}{\partial t}$ is the serpentinization rate of a given model mesh cell (unit: s^−1^) and *T* is the temperature in degrees Celsius of that cell, while empirically fitted kinetic coefficients (*A* = 10^−10^ s^−1^, *b*_s_ = 2.5 × 10^−4^°C^−2^, *c*_s_ = 270°C) have been derived from experimental and theoretical observations on serpentinization kinematics (*18*).

With the serpentinization rate, we can calculate the serpentinization capacity (*M*_t_) along the modelled section using the following equation from (*12*) in the ParaView Python calculator

$$M_{t}=\sum_{i=1}^{i=\text{ncell}}\left(\frac{\partial \text{Dserp}}{\partial t}\right)\left(i\right)\;S_{i}\;\rho_{m}$$ (8)

where $\left(\frac{\partial \text{Dserp}}{\partial t}\right)\left(i\right)$ is the serpentinization rate of a given model mesh cell *i*, with *S_i_* being the area of that cell, and ρ_m_ the density of the material in that cell. By summing over all cells (ncell), and given that our geodynamic ASPECT model is 2D, we obtain *M*_t_ in units of kg(m s)^−1^, which we convert to kg (km year)^−1^. Here, the “km” indicates length along-strike of the tectonic system (i.e., perpendicular to the 2D geodynamic ASPECT model; Figs. 2 and 7).

We subsequently derive the natural H_2_ capacity in units of mol(km year)^−1^, assuming that 1 kg of serpentinized mantle material generates between 100 and 300 mmol of natural H_2_ (*11*). We thus obtain an upper limit estimate of the natural H_2_ capacity in our modelled rift-inversion orogens while accounting for the considerable variations in natural H_2_ generation potential that may occur in natural systems (*11*).

### Nuances to our model analysis

We must consider some nuances to our first-order modeling approach, which involves a number of assumptions and simplifications required for the models to be realized, as specified below. We believe that our results and the orogenic natural H_2_ system concept (Fig. 5) we present in this study are robust, providing key insights and a solid framework for understanding natural H_2_ potential in rift-inversion orogens. Still, they should not be directly applied to interpret complex natural cases but rather serve as inspiration for more detailed assessments while keeping in mind the below nuances.

#### Large-scale limitations to the geodynamic models

Our models are essentially 2D (Fig. 2), whereas natural orogens are complex 3D structures [e.g., (*71*)], each with a unique history that requires assessment of its natural H_2_ potential on a case-by-case basis, with an eye on variations along the strike of the system. Even so, the general structures we obtain in our models are realistic on a first-order basis (Figs. 1, 2, and 6). Moreover, because of constraints in the ASPECT code, melting and oceanic crust formation are not included in our models. However, we do develop the thermal profile that would be expected from an oceanic basin [Fig. 2, C and G, and (*37*)], whereas the thin sediment cover developing in rift basins provides a brittle layer that behaves in a similar way to oceanic crust. In addition, the mafic oceanic crust can also serpentinize, in a similar way as the mantle material exhumed in our modeled rift basins. Another caveat is that our analysis does not allow the detailed tracing of serpentinized and unserpentinized (fresh) mantle rock, so that some overlap and thus overestimation of serpentinized mantle volumes may occur. Still, our models suggest general exhumation of fresh mantle material during rift-inversion orogen evolution: by mantle uprising below the rift basin during rifting and by overthrusting of the overriding mantle wedge during inversion (Fig. 3).

#### Extent of the serpentinization window and omittance of subduction-related serpentinization

For our natural H_2_ potential analysis, we focus on serpentinization of exhumed mantle material, which can be considered to be most efficient at temperatures between 200° and 350°C during rifting (*12*, *18*). To ensure consistency in our analysis, we apply this same window to assess the natural H_2_ potential throughout the whole evolution of the rift-inversion orogens. Yet, this serpentinization window is rather conservative when considering the inversion stage of rift-inversion evolution because efficient serpentinization can also occur at higher temperatures and pressures (*19*, *23*). We conducted a test with a more extensive serpentinization window of 200° to 500°C that may be more representative for deeper (10 km) serpentinization during inversion (*19*). We find that, although there would be more serpentinization in the system overall, the ratio of serpentinization per time unit between rifting and inversion would remain similar or would even be larger when applying these different windows for rifting and inversion, respectively [see details in (*37*)]. Regardless of how the serpentinization window is parametrized, the fact that orogens are colder than rifts means that the orogenic serpentinization window is decisively larger and hence that considerably more natural H_2_ is generated.

Moreover, because we focus our analysis on exhumed mantle material (i.e., in the overriding mantle wedge in a rift-inversion orogen; Fig. 1C), we omit the potential additional serpentinization and natural H_2_ generation in the deepest parts of the system during inversion (at depths of 50 to 60 km in the subduction channel) (*19*, *23*). Including this deep inversion-related serpentinization, a challenge beyond the scope of our study, would increase the already distinct difference in serpentinization between rifting and inversion.

#### Water availability

Liquid water is key to natural H_2_ generation because serpentinization cannot occur without it. In our analysis, we assume that liquid water, either from surface (marine or meteoric) or from deep sources (metamorphic), is readily available along large active faults that cross exhumed mantle material. This assumption is reasonable during rifting, when seawater can percolate along large normal faults. The situation is more complex in rift-inversion orogens, where meteoric water may reach deep rocks as a result of the high hydraulic head and temperature differences (*21*), whereas dehydration reactions linked to subduction may release large volumes of water that can induce serpentinization in the overlying mantle rocks as well [e.g., (*23*) (Fig. 5)]. A thorough understanding of fluid flow and water-rock reactions is critical for understanding the spatial and temporal distribution of serpentinization in natural systems. For example, the impact of density changes during serpentinization requires attention because the volumetric expansion of serpentinizing mantle material may either clog water pathways (*72*) or induce new fracturing and increase permeability (*73*). Still, we can expect large-scale faults to generally provide efficient pathways for water circulation, both in rifting and inversion settings.

#### Quantities of generated natural H_2_

Serpentinization cannot directly be equated to H_2_ generation since next to temperature, pressure and water availability, the type (composition) and freshness of mantle material, as well as the origin and associated chemistry of the water involved in serpentinization modulate natural H_2_ generation potential (*11*, *40*, *68*). As a result, serpentinization reactions are very complex, and corresponding natural H_2_ generation from mantle material has a considerable range in nature. We account for this uncertainty by applying a range of 100 to 300 mmol of natural H_2_ per kilogram of mantle rock in our first-order natural H_2_ potential analysis (*11*).

#### Natural H_2_ migration and preservation

Other limitations to our models concern the omission of detailed sedimentary history (i.e., timing and availability of reservoirs and seals) and of detailed natural H_2_ migration pathways to potential reservoirs because highest model resolution of 625 m only allows the tracing of the largest faults [e.g., (*21*, *25*)]. Rock-water interactions and the aforementioned volumetric expansion of serpentinizing mantle material and impact on fault permeability (either increasing or decreasing) also affect the efficiency of natural H_2_ migration pathways (*72*, *73*), although the size of H_2_ molecules means that they can move more easily along tight passages than other substances. These reactions are also relevant for the quality of reservoir rocks and seals. A further important question is whether the high buoyancy of H_2_ molecules allows them to move upward with relative freedom, or whether their migration will be meaningfully affected and redirected by fluid flows along major faults.

Moreover, H_2_ gas is highly reactive and may easily be consumed by (bio)chemical reactions during serpentinization, as well as on its way to, or within reservoirs [e.g., (*8*, *74*)]. For example, during or shortly after serpentinization, the resulting natural H_2_ can be swiftly converted to CH_4_ or other by-products (*23*, *51*), which may subsequently end up in the reservoirs that would otherwise accumulate the natural H_2_. Last, although we point out that reservoir temperatures of natural H_2_ fields should ideally be higher than the 122°C biotic fringe at several kilometer depth for the best preservation conditions (*20*, *40*), much shallower natural H_2_ accumulations at some hundreds of meters depth are known from Mali and Albania (*16*, *33*), potentially due to very high H_2_ fluxes that replenish any loss of H_2_.
